# College Students’ Distractions from Learning Caused by Multitasking in Online vs. Face-to-Face Classes: A Case Study at a Public University in Romania

**DOI:** 10.3390/ijerph191811188

**Published:** 2022-09-06

**Authors:** Kamer Ainur Aivaz, Daniel Teodorescu

**Affiliations:** 1 Department of Economics, Ovidius University of Constanta, 900527 Constanta, Romania; 2 Department of Educational Leadership, School of Education, Clark Atlanta University, Atlanta, GA 30314, USA

**Keywords:** multitasking, digital distractions, online education, face-to-face classes, impact on learning

## Abstract

This study compared Romanian college students’ off-task use of digital devices in online courses with their digital distractions in face-to-face courses. Students taking both online and face-to-face courses completed surveys assessing off-task use of technology in each delivery mode, extent to which such use constitutes a distraction, and instructor policies for curbing use of digital devices in class. Off-task use of digital devices was significantly greater in online than face-to-face courses. Students were twice as likely to state that they were distracted by multitasking in online than in face-to-face classes. They also indicated that instructors in online classes were less likely than their face-to-face instructors to have a policy on the use of digital devices for off-task purposes during class. Study participants were more lenient toward the use of digital devices in online than in in-person classes and were less bothered by multitasking and classmates’ off-task use of digital devices in online than in face-to-face classes. The results of this study have implications for instructors in online classes. By improving instructional design and pedagogical methods and creating opportunities for more interaction during the class, online instructors might be able to increase student motivation and lower multitasking behaviors.

## 1. Introduction

Today’s college students live in a complex world, a world full of distractions and interruptions caused by social media that may prevent them from achieving their educational goals. The term “interference,” which includes distractions and interruptions, is commonly used to describe something that slows down, obstructs, impedes, and largely diverts another process. Interferences are generated both internally, in the form of thoughts that appear unexpectedly in our mind, and externally, due to sensory stimuli such as noise, beeps, vibrations, light signals produced by digital devices [1]. Distractions are fragments of information irrelevant to our tasks, which we either encounter outside or generate ourselves inside our mind, and interruptions occur when one makes the decision to engage in more than one task at a time. Interruptions in which people try to perform two or more tasks simultaneously aimed at goals that are independent of each other, are often referred to as multitasking [2]. College students are facing an explosion in the variety and accessibility of technologies with enticing sounds, pleasing visual effects and insistent vibrations that demand their attention, while their brains are battling multiple streams of information at the same time [2], thus affecting learning.

The goal of this paper is to add to the discussion of the impact of distractions on learning by examining how college students at a Romanian public university perceive the off-task (unrelated to school) use of technology in class. This behavior has often been referred to as “cyberslacking.” Analyzing data from student surveys, we examine (1) how often students use digital devices in class (online vs. in person) for off-task purposes, (2) the extent to which students perceive the off-task use of technology as a distraction, and (3) what instructors and universities should do to minimize off-task technology use in class.

### Digital Distractions in Higher Education

The new social media applications have created a permanent change in the adolescents and young adults’ capacity to focus. Smartphones, desktops, and laptops support countless applications, while browsers allow many windows to be open, making it extremely difficult for them to focus on a single site or application without being distracted by another site/application. Additionally, there is a growing tendency to multitask, particularly while online. A study by Carrier et al. [3] showed that a teenager can handle six or seven forms of media at the same time. Adults are affected as well. Other studies have shown that up to 95% of the population reports a daily access to several environments at the same time, and this type of multitasking activity occupies them about a quarter of the day [4,5,6]. Nielsen researchers [7] monitored the use of mobile phones by 3743 American adults to examine their motivations for using apps and concluded that 70% use them when they are alone, 68% when they are bored, and 61% while waiting for something or someone. Ahonen [8] showed that adults and adolescents check their phone up to 150 times a day, that is, every six or seven minutes.

Similar studies in the U.K. revealed that more than half of adults and teenagers do not let an hour pass without checking their phone. According to Harris Interactive, three of four mobile users feel panicked when they cannot find their phone right away, one in two check their phone messages as soon as they wake up, while they are still in bed, and one in three check it in the bathroom, while three in ten check it when they dine with other people [9].

The permanent accessibility, along with invasive notifications and the encouragement to transition from one task to another have generated a higher level of interference than we would have thought. However, while we are beginning to realize that these interferences often hinder our achievement of goals and tasks, most of us adopt interfering behaviors even when distractions and multitasking could be completely avoided [2]. However, why do we do this? A common explanation is that we have more fun and enjoy more rewards if we multitask than if we focus on a single task [10]. Related to the rewards of multitasking, researchers have shown that novelty is associated by our brains with reward processing [11]. Another hypothesis, explored by Gazzaley and Rosen [2], is that we adopt behaviors that cause interference because, from an evolutionary point of view, we behave optimally when we satisfy our innate tendency to seek information, and the technological world in which we live offers us many opportunities to nurture this tendency and to influence boredom and fatigue. Thus, the search for informational food engages us in behaviors that are meant to increase our exposure to and consumption of new information, even if it leads to interference [12].

There is evidence that both the boredom and anxiety levels we experience as we search for information are increasing in direct proportion to the scale of modern technology. We get bored of what we do and are more impatient than before and we often move our attention to another place. Eastwood et al. [13] noted that boredom occurs when: we are not able to successfully focus on something due to external environmental thoughts, feelings, or stimuli; when we are convinced that we are not able to focus and participate satisfactorily in a particular activity; and when we attribute the cause of our distraction to the environment.

The effects of off-task use of technology by college students in the class have been explored in recent years by many educational researchers. Nieterman and Zaza [14], for instance, revealed that while students at a Canadian university acknowledged that the off-task technology use can be distracting, they considered it a matter of personal autonomy, which should only be regulated when it creates distractions for others. Others noted that the temptation caused by the proliferation of social media platforms and use of digital devices for purposes unrelated to class and for multitasking has resulted in lower academic performance and lower educational satisfaction and has negatively affected the student–instructor rapport [15,16,17].

Risko et al. [18] demonstrated that using technology for both class-related and class-unrelated purposes can result in cognitive overload (decline in the ability to retain and process the information) during lectures while Junco and Cotton [19] showed that multitasking can lead to a decline in students’ academic performance. Additionally, research findings also indicated that off-task use of technology affects more the academic performance of lower-achieving students, who also have less capacity for self-control and self-regulation than their better-achieving peers [20,21].

More recently, Epp et al. [22] conducted a study of students taking both online and face-to-face courses at a large, public university in the Midwestern region of the United States. The authors found that multitasking was significantly greater in online than in face-to-face courses. Additionally, there were different sets of predictors for students’ multitasking in online courses compared with face-to-face courses. The authors inferred that multitasking in online and face-to-face courses are different phenomena, and therefore may require different pedagogical approaches to successfully minimize multitasking behaviors.

The literature also shows that it is not clear what universities can do to curb multitasking and remedy the negative impact of off-task use of technology on learning. Given the rapid integration of technology into everyday life and college classes, higher education instructors might not be able to enforce institution-wide bans [23], although such bans have been tried in secondary education systems [20]. As Nieterman and Zaza [14] noted, digital devices can provide access to education for students with disabilities, making such bans discriminatory and in violation of legislation regarding students with disabilities.

Off-task use of digital devices in class might be explained in part by low teaching effectiveness and lack of intellectually engaging sessions. For instance, it has been shown that students’ lack of motivation in online classes is associated with low instructor effectiveness and low use of interactive tools [24]. Similarly, students taking online classes during the pandemic reported low levels of interaction and did not participate in collaborative learning, which adversely impacted the development of their social skills [25,26]. The low level of interactivity in online education affected students in medical fields more than other students [27].

While the existing research clearly demonstrates the negative impact of off-task use of technology on learning, we do not know if the difference in multitasking between online and face-to-face found in the United States and Canada also holds for students in less developed countries, including Romania. This is significant because the higher education system in Romania has recently witnessed a growth in online and hybrid programs during the COVID-19 pandemic. If the findings from the developed world hold true, the explosion of hybrid and online classes following the outbreak of COVID-19 further exacerbates the off-task use of digital devices and their impact on learning in the Romanian higher education system.

Therefore, the purpose of this study is to examine: (1) how often Romanian students at a select public university use digital devices in class (online vs. in person) for off-task purposes; (2) the extent to which Romanian students perceive the off-task use of technology to be a distraction in online vs. in-person classes; and (3) what the Romanian students’ perceptions are as to what instructors and universities should do to minimize off-task technology use in face-to-face vs. online classes. Given the established tendency of students in developed countries to multitask more in online than in face-to-face courses [22], the current study hypothesizes that Romanian college students are also more likely to multitask in online than in face-to-face courses.

## 2. Materials and Methods

### 2.1. Instrumentation

The questionnaire we developed for this study was inspired by the findings reported by Nieterman and Zaza [14] on students’ and instructors’ perspectives on off-task use of technology in the classroom. The study was conducted in 2016–2017 at the University of Waterloo, ON, Canada in the Faculty of Applied Health Sciences.

The focus of our questionnaire was students’ off-task use of technology in class during the COVID-19 pandemic, perceptions regarding the extent to which it may hinder learning and opinions as to what instructors should do to minimize technology-related distractions in class (see Appendix A). Most questions used 5-point Likert scales as response options. Students were asked to share their perceptions related to off-task use of technology in both online and in person classes.

### 2.2. The Research Setting

The research setting for this study was Ovidius University, which is the successor of the first higher education institution in Constanța, the Pedagogical Institute of Constanța, founded in 1966. In 1990, it became a multidisciplinary university that offers bachelor’s, master’s and doctoral programs, and it is recognized by national and international accreditation bodies. It is currently the largest European university on the Black Sea coast. In Fall 2020, about 15,000 students were studying at Ovidius University, guided by 650 instructors and researchers, and supported by over 300 staff members and administrators. The university is multidisciplinary, educating students in degree programs that span all study cycles, from bachelor’s to master’s and doctorate, as well as various pedagogical training courses, professional development courses for certified teachers, and medical residency programs. The fields of study offered cover a wide range, including medicine, engineering, humanities, natural sciences, social sciences, law, arts, and theology. The university currently has 86 undergraduate programs in 44 fields, 76 master’s programs, and four doctoral schools in eight fields of study. The university is organized in 16 schools (faculties) and there are 487 students enrolled in the doctoral programs and 870 medical residents.

### 2.3. Description of the Sample

For this study, the population of interest was only undergraduate students enrolled in the Faculty of Economic Studies at Ovidius University (*N* = 1930). We employed a convenience sample of 630, yielding a response rate of 33%. The sample excluded students enrolled in distance education programs. The demographic profile of the participants in this study is shown in Table 1. The large majority of respondents were female students (77.5%), in their first year of study (59.5%), and not employed (54.4%). All three years of study were represented in the sample: 59% were in 1st year, 24% were in second year, and 17% were in third year. As the age breakdown shows, most participants were either adolescents (age < 19) or young adults (in their twenties).

### 2.4. Data Collection Procedures

The researchers obtained approval from the Deputy Rector for Education and Quality Management at Ovidius University to conduct the study. Participants were recruited during class time in face-to-face courses and were emailed the link to the survey developed in Qualtrics. The first page of the survey served as a consent form, informing students of the purpose of the study and ensuring that all responses to the survey will remain anonymous and that results will be reported in aggregated form. Participants were also informed that participation in the study was voluntary and that they could withdraw from the study at any time without repercussions. Data collection lasted between 26 May 2021 and 7 June 2021. At the time of the survey, the majority of study participants had experienced both online and in person classes and were able to compare their use of digital devices. However, given the lockdown caused by the COVID-19 pandemic, there were more students in the sample, especially among first-year students, who experienced only online classes than students who took in-person classes.

### 2.5. Data Analysis

The survey data were analyzed using SPSS 28 (IBM Corp., Armonk, NY, USA). The main objective of the analysis was to conduct a comparative study of two groups of answers: “Online Courses,” where we included student answers referring to instruction conducted in the virtual environment, and “In Person Courses,” which included answers regarding face-to-face instruction. Pearson’s Chi Square statistics were used to explore significant differences between the two groups of responses for a range of questions related to digital distractions. Cross-tabulations were also conducted to examine the use of digital devices by gender, age, employment status, and year in college. The results of these cross-tabulations are reported in Section 4.

## 3. Results

Participants were asked to indicate how often in a typical day they used their digital devices (i.e., laptops, smartphones, computers, tablets) during online and in-person courses for other than course-related activities (i.e., texting, browsing the Internet, posting to social media). It should be noted that not all participants had experienced both online and in-person classes, and therefore the total number of respondents was lower than 630 for some of the survey items. There was a significant difference between the two modes of instruction, with 95% of students saying that they checked their devices in online courses for off-task purposes, compared to 75% in face-to-face classes, χ^2^ (1) = 148.33, *p* < 0.001. Approximately 15% said that they checked their devices more than 30 times during online classes (Table 2).

When asked about the main reason they use their digital devices during class for off-task purposes, there were significant differences in behaviors between online and in-person classes (Table 3). Although the majority of students used their devices to check time in both delivery modes, students were more likely to use their devices to read/send emails or texts in online classes than in in-person classes (25.4% vs. 13.3%).

Participants were also asked to indicate what percentage of the class time they spent using their devices for reasons not related to school (off-task purposes). About 44% in online and 37% in face-to-face classes spent more than 20% of class time using their devices. The difference was statistically significant at the 0.001 level, with students reporting more frequent use of their devices in online than in face-to-face classes (Table 4).

Approximately one-fourth of the students said that they were not distracted when they used their device during class, and this varied considerably between online and in person classes. As Table 5 shows, more students reported that they were distracted “a lot” by their off-task use of devices in face-to-face (11.3%) than in online classes (7.1%). All online classes offered at the examined institution were synchronous, where all students must attend at once.

When classmates use their digital devices in class for non-school related purposes, students were more distracted by this behavior in in-person than in online classes. About 70% said that the off-task use of devices by other students in face-to-face classes distracted them, compared to 42% in online classes (Table 6).

In both delivery modes, about half of the participants stated that, when they were distracted by classmates, they were bothered mostly by audio distractions such as alerts, music, and conversations (Table 7). However, in online classes, students were more likely to be bothered by visual distractions than in in-person classes (25.1% vs. 19.6%). This might be explained by the fact that students can see images in Zoom of their classmates (if they have the camera turned on) being preoccupied with off-task activities.

When speaking of their experience in online classes, only 34% of participants stated that their professors established a policy on the use of digital devices during class (see Table 8). For in-person classes, students were two times more likely to report that their instructors had such a policy (70%). The difference in responses between the two delivery modes was significant at the 0.001 level.

When referring to the in-person classes they had enrolled in, approximately half of the respondents (48.4%) said that they did not use digital devices during classes because they distract them from learning. However, only 21.2% said the same of their online classes. Related to online classes, more than one-third of respondents used their devices during class for reasons unrelated to school and 29.4% considered it to be their choice to use their digital devices any time they wanted (Table 9).

When asked whether universities need to introduce policies that restrict the use of digital devices in class, there was a significant difference between the responses related to online and in-person classes. More than half of participants (53.7%) thought that such policies were needed for in-person classes, but only 38.3% considered them necessary for online classes (Table 10).

Approximately six in ten students would like instructors to ban the use of digital devices during in-person classes. However, only two in 10 students considered that this ban is needed for online classes. The difference was statistically significant at the 0.001 level (Table 11).

Participants were also asked to indicate how instructors should react when they see that a student in their class is using a digital device for reasons unrelated to school. Referring to online classes, about one-third of the respondents considered that students should receive only a warning after the first offense, but they should be punished after each other subsequent offense. Only 10.5% thought that students should be punished after each offense, including the first offense. About one-fourth (23.7%) of the respondents stated that instructors should not take any punitive measure against students’ off-task use of technology.

Overall, students were less lenient in their attitudes toward the off-task use of digital devices in online than in in-person classes. About 22% would like the off-task use of digital devices in in-person classes to be punished every time it is detected, compared to 10.5% for online classes (Table 12). Students were more likely to say that no punitive measure should be taken in online (23.7%) than in face-to-face classes (18.4%).

Survey participants were asked to indicate specific actions professors should take when a student distracts the class by using a digital device for off-task purposes. Again, students were more lenient toward such incidents in online than in in-person classes. About three-fourths said that the instructor should talk to the student in online vs. 46.1% in in-person classes. Additionally, more respondents indicated that the instructor should ask the student to leave the class in in-person courses than in online courses (Table 13).

## 4. Discussion

Today’s college students were raised during a period of social media explosion and spent most of their time in multitasking mode. As such, they face a major challenge staying focused during their academic studies. When they come to college, they already have the predisposition to consult multiple sources of information and multitask on their digital devices. As Rosen et al. [21] noted, the average student could not concentrate for more than 3–5 min, and a single interruption, such as browsing through the Facebook feed, was enough to predict poor academic results. Therefore, lack of focus during online classes can have disastrous consequences for a student’s grades and prospects for graduation. Additionally, Epp et al. [22] showed that multitasking was significantly greater in online than face-to-face courses for undergraduates in the U.S.A. Similarly, Moreno at al. [27] concluded that undergraduate college students are frequent multitaskers, particularly in online classes.

The lockdown triggered by the COVID-19 pandemic has fundamentally changed the behaviors of postsecondary students, as they faced physical isolation from peers and instructors for extended periods of time. Most likely, the lockdown increased the frequency of off-task use of digital devices during class and study time, which has influenced students’ responses to our survey. In discussing the results related to use of digital devices in class for purposes not related to school, it is instructive to examine use by gender, employment status, age, and year in college. In both in-person and online classes, female students were more likely to exhibit heavy use of their digital devices (more than 10 times during classes per day) than male students (17.1% vs. 12.4% in in-person classes, and 42.4% vs. 27.6% in online classes).

In in-person classes, students who worked were also more likely to show excessive multitasking behaviors than students who did not work (full-time employed, 17.9%; part-time employed, 22.4%; and not employed, 13.2%). However, this finding did not hold for online classes (full-time employed, 35.4%; part-time employed, 42.9%; not employed, 40.5%).

It appears that excessive use of digital devices decreases with age in in-person classes: 20.4% for 19 year olds, 17.0% for 20 year olds and 9.4% for 21 year olds. In online classes, the percentage of heavy users across the three age groups were 44.4%, 36.7%, and 37.9%, respectively. In in-person classes, first-year students (18.9%) were more likely to be heavy off-task users than students in their second or third year (12.1%). In online classes, the percentage of excessive off-task users was 38.2% for first year, 42.4% for second year, and 36.7% for third year students.

This study provides further evidence in support of the study published by Epp at al. [22] and other studies conducted in the developed world and shows that Romanian students also tend to multitask significantly more in online than in face-to-face classes. Additionally, students have different attitudes toward multitasking in online than in face-to-face classes, although the impact of distractions on their learning might be comparable. Students in face-to-face classes are twice more likely than students in online classes to say that digital devices distract them from learning. Generally, students also are more lenient toward the use of digital devices in online than in in-person classes and are less bothered by multitasking and classmates’ off-task use of digital devices in online classes than in face-to-face classes. After all, in an online class, the use of digital devices for off-task purposes is not necessarily visible or audible, and not all students will have their cameras turned on during a session.

Iluzada et al. [28] found that students tend not to favor restrictive personal technology policies. Similarly, this study shows that most students reject policies for controlling use of devices in class. Therefore, instructors should consider other approaches to curbing digital distractions in the classroom such as using mobile technology as a teaching tool, teaching students to be self-regulated learners, and motivating students to delay gratification from their mobile devices [29].

Additionally, this study showed that students tend to suggest more punitive actions for the off-task use of digital devices in in-person than in online classes. Corroborating the findings obtained for the Canadian students [14], the Romanian students in this study viewed multitasking in class as a matter of individual freedom, personal decision-making, and personal autonomy. Approximately 29% of the students considered that it was their personal choice to use their digital devices any time they want in online classes. This finding implies that an institution-wide ban on off-task use of digital devices might be difficult to enforce, especially in online classes. More than half of participants (53.7%) thought that such policies were needed for in-person classes, but only 38.3% considered them necessary for online classes. Additionally, approximately six in ten students would like instructors to ban digital devices during in-person classes, but only two in 10 students considered that such a ban was necessary for online classes. However, confiscating the digital device may pose legal issues and would be difficult to enforce. Some students might have mobile devices with apps for disabilities. Other students might use their devices for on-task and lecture-related purposes. Given that most instructors allow use of digital devices in class, it is important that they explore ways in which students can integrate the use of such devices with teaching and learning [30]. As Migdalski [31] noted, smartphone technology possesses the capabilities of information search, video and audio recording, instant communication, and a variety of applications which, if properly used, can serve to enhance the teaching/learning environment both inside and outside the classroom.

## 5. Conclusions

The results of this study could influence the expected growth in online or hybrid higher education in Romania and have implications for instructors as they design and conduct their online classes. By improving instructional design and pedagogical methods, and by creating opportunities for interaction during the class and offering intellectually engaging sessions, online instructors might be able to lower their students’ multitasking behaviors. As shown by earlier studies [24,32], poor teaching effectiveness leads to lower student satisfaction with the class and lack of motivation to succeed in the class, which can in turn trigger cyberslacking. Additionally, higher education institutions need to develop policies to help their instructors limit the off-task use of technology in online classes, particularly in face-to-face classes.

Future qualitative research should investigate why students choose to multi-task during class. It could be that students are making sensible and reasonable decisions about how best to use their time and devices in dealing with complex lives, especially those students that work while in college. However, most of the research on cyberslacking tends to focus on the deleterious influence digital distractions have on the integrity of the classroom learning environment, as perceived by instructors [33], and does not sufficiently explore the positive aspects of multi-tasking.

There are several limitations associated with the design of this study. First, the convenience sample limited to economics students does not allow the researchers to extrapolate the results to students in other fields or other universities within the Romanian higher education system. Second, not all of the students in the sample took both online and in-person classes; there were more students who had experienced only online classes, since they started their program during the lockdown. Third, the study used only self-reported data, which might not yield highly reliable estimates for the off-task student use of digital devices. Fourth, descriptive studies are limited in that they cannot be used to establish cause and effect relationships, respondents may not be truthful when answering survey questions or may give socially desirable responses, and the choice and wording of questions in the questionnaire may influence the descriptive results (i.e., means and percentages).

Despite its limitations, this paper addresses an important gap in the existing literature on digital distraction in the college classroom. While most studies on cyberslacking focus on research settings in the Western world, this study demonstrates that the behaviors and attitudes toward the use of digital devices exhibited by college students in Romania share many similarities with those of students in Western countries.

## Figures and Tables

**Table 1:** Sample demographics.

	*n*	*%*
*Field of Study*		
Economics of Commerce, Tourism and Services	166	26.3%
Microeconomics	1	0.2%
Business Management (in English)	14	2.2%
International Affairs	25	4.0%
Accounting and Management Information Systems	94	14.9%
Finance and Banking	144	22.9%
Marketing	78	12.4%
Management	108	17.1%
Total	630	100.0%
*Year of Study*		
1	375	59.5%
2	151	24.0%
3	104	16.5%
Total	630	100.0%
*Gender*		
Male	142	22.5%
Female	488	77.5%
Total	630	100.0%
*Age*		
19 or younger	174	27.6%
20	183	29.0%
21	138	21.9%
22	47	7.5%
23	19	3.0%
24	19	3.0%
25 or older	50	8.0%
Total	630	100.0%
*Employment status*		
Employed full-time	218	34.6%
Employed part-time	69	11.0%
Not employed	343	54.4%
Total	630	100.0%

**Table 2:** In a typical school day, how often do you use your digital devices during your classes for other than course-related activities?

	Online Courses		In-Person Courses	
	*n*	*%*	*n*	*%*
Never	28	4.9%	87	24.3%
1–3 times	141	24.9%	113	31.6%
4–10 times	176	31.1%	101	28.2%
11–30 times	135	23.9%	57	15.9%
More than 30 times	86	15.2%	0	0.0%
Total	566	100.0%	358	100.0%

χ^2^ (1) = 148.33, *p* < 0.001.

**Table 3:** If you use your digital device for other than course-related activities, what is the main reason for doing that?

	Online Classes		In-Person Classes	
	*n*	*%*	*n*	*%*
To check other people’s posts or post myself on social media	50	8.7%	25	6.8%
To read or send emails or text	146	25.4%	49	13.3%
To surf the Internet	59	10.3%	22	6.0%
To check the time	237	41.3%	194	52.7%
I do not use digital devices during classes	82	14.3%	78	21.2%
Total	574	100.0%	368	100.0%

χ^2^ (1) = 244.48, *p* < 0.001.

**Table 4:** If you use your digital device for reasons not related to school during class, what percentage of class time do you spend on your devices?

	Online Classes		In-Person Classes	
	*n*	*%*	*n*	*%*
I do not use digital devices during classes	83	14.9%	97	26.9%
1–20%	231	41.5%	165	45.7%
21–40%	121	21.7%	53	14.7%
41–60%	72	12.9%	30	8.3%
61–80%	50	9.0%	16	4.4%
Total	557	100.0%	361	100.0%

χ^2^ (1) = 222.55, *p* < 0.001.

**Table 5:** How distracted are you when you use digital devices during class for reasons not related to school?

	Online Classes		In-Person Classes	
	*n*	*%*	*n*	*%*
I am not distracted at all	113	23.7%	67	23.8%
It distracts me a little	196	41.1%	103	36.5%
It distracts me somewhat	76	15.9%	47	16.7%
It distracts me a lot	34	7.1%	32	11.3%
I cannot focus at all	58	12.2%	33	11.7%
Total	477	100.0%	282	100.0%

χ^2^ (1) = 159.14, *p* < 0.001.

**Table 6:** How distracted are you when your classmates use their digital devices during class for reasons not related to school?

	Online Classes		In-Person Classes	
	*n*	*%*	*n*	*%*
I am not distracted at all	263	57.9%	74	29.1%
It distracts me a little	103	22.7%	116	45.7%
It distracts me somewhat	35	7.7%	27	10.6%
It distracts me a lot	19	4.2%	22	8.7%
I cannot focus at all	34	7.5%	15	5.9%
Total	454	100.0%	254	100.0%

χ^2^ (1) = 150.63, *p* < 0.001.

**Table 7:** What type of distraction bothers you the most during class?

	Online Classes		In-Person Classes	
	*n*	*%*	*n*	*%*
Visual distractions (e.g., Internet surfing)	112	25.1%	47	19.6%
Audio distractions (e.g., alerts, music, conversations, etc.)	223	49.9%	125	52.1%
It does not bother me	112	25.1%	68	28.3%
Total	447	100.0%	240	100.0%

χ^2^ (1) = 115.21, *p* < 0.001.

**Table 8:** Have your professors introduced a course policy regarding the use of digital devices during class?

	Online Classes		In-Person Classes	
	*n*	*%*	*n*	*%*
Yes, established policy	150	33.8%	151	70.2%
No	294	66.2%	64	29.8%
Total	444	100.0%	215	100.0%

χ^2^ (1) = 150.00, *p* < 0.001.

**Table 9:** Which of the following statements best describes your use of digital devices for other than course-related activities?

	Online Classes		In-Person Classes	
	*n*	*%*	*n*	*%*
I can use digital devices for reasons unrelated to school.	161	34.9%	38	17.8%
It is my choice to use digital devices any time I want.	136	29.4%	40	18.8%
I do not use digital devices during classes because they distract me from learning.	100	21.6%	103	48.4%
I cannot stop using digital devices during class even though I know they distract me.	65	14.1%	32	15.0%
Total	462	100.0%	213	100.0%

χ^2^ (1) = 90.55, *p* < 0.001.

**Table 10:** Do you think universities need to introduce policies that limit students’ use of digital devices during class?

	Online Classes		In-Person Classes	
	*n*	*%*	*n*	*%*
Yes	176	38.3%	87	53.7%
No	283	61.7%	75	46.3%
Total	459	100.0%	162	100.0%

χ^2^ (1) = 118.00, *p* < 0.001.

**Table 11:** Should instructors ban the use of digital devices during class?

	Online Classes		In-Person Classes	
	*n*	*%*	*n*	*%*
Yes	87	20.0%	102	58.6%
No	349	80.0%	72	41.4%
Total	436	100.0%	174	100.0%

χ^2^ (1) = 107.00, *p* < 0.001.

**Table 12:** What measures should be taken against a student who uses digital devices during class for reasons unrelated to school?

	Online Classes		In-Person Classes	
	*n*	*%*	*n*	*%*
Warning after the first offense, and punishment after subsequent offenses.	270	65.9%	110	59.4%
Punishment after each offense.	43	10.5%	41	22.2%
No measure should be taken.	97	23.7%	34	18.4%
Total	410	100.0%	185	100.0%

χ^2^ (1) = 48.33, *p* < 0.001.

**Table 13:** What should a professor do to a student who disturbs the class by using a digital device?

	Online classes		In-Person Classes	
	*n*	*%*	*n*	*%*
Talk to the student	309	74.6%	89	46.1%
Ask the student to leave the class	76	18.4%	57	29.5%
Confiscate the student’s digital device	29	7.0%	47	24.4%
Total	414	100.0%	193	100.0%

χ^2^ (1) = 55.93, *p* < 0.001.

## Data Availability

Not applicable.

## Appendix A. Digital Distractions Survey

**1. Field of study:**

◯ Economics of Commerce, Tourism and Services
◯ Microeconomics
◯ Business Management (in English)
◯ International Affairs
◯ Accounting and Management Information Systems
◯ Finance & Banking
◯ Marketing
◯ Management

**2. Year of study in the program:**

◯ 1st
◯ 2nd
◯ 3rd

**3. Your gender:**

◯ Male
◯ Female

**4. Your Age: __________**

**5. Your employment status:**

◯ Full-time employed
◯ Part-time employed
◯ Unemployed

**6. In a typical school day, how often do you use digital devices (smartphone, laptop, computer) in your classes for other than course-related activities (i.e., sending messages, surfing the Internet, checking or posting on social media)?**

▪	Online Classes	In Person Classes
▪ Never	▢	▢
▪ 1–3 times	▢	▢
▪ 4–10 times	▢	▢
▪ 11–30 times	▢	▢
▪ More than 30 times	▢	▢

**7. If you use a digital device during class for tasks that are not related to school, what is your main reason for doing that?**

▪	Online Classes	In Person Classes
▪ To check other people’ posts on social media or to post	▢	▢
▪ To read or send an email or text	▢	▢
▪ To surf the Internet	▢	▢
▪ To check the time	▢	▢
▪ I do not use digital devices during class	▢	▢

**8. If you use a digital device during class for tasks that are not related to school, approximately what percentage of the class time do you allocate to using your digital device?**

▪	Online Classes	In Person Classes
▪ I do not use digital devices during class	▢	▢
▪ 0–20%	▢	▢
▪ 21–40%	▢	▢
▪ 41–60%	▢	▢
▪ 61–80%	▢	▢
▪ 81%+	▢	▢

**9. What is the main benefit of using your digital device during class for reasons not related to school?**

◯ I am permanently connected
◯ I am having fun
◯ I avoid boredom
◯ I am ready in case there is an emergency
◯ My use of the device is related to tasks in class

**10. What is the main disadvantage of using your digital device during class for reasons not related to school?**

◯ Distracting my classmates
◯ Losing the information presented in class
◯ Getting poor grades on exams
◯ The possibility of being noticed the instructor and asked about the information presented in class
◯ Losing the ability to focus

**11. How distracted are you when you use a digital device in class for reasons not related to school?**

▪	Online Classes	In Person Classes
▪ I am not distracted at all	◯	◯
▪ It distracts me a little	◯	◯
▪ It distracts me some	◯	◯
▪ It distracts me a lot	◯	◯
▪ I cannot focus at all	◯	◯

**12. How distracted are you when your classmates use their digital devices during class for reasons not related to school?**

▪	Online Classes	In Person Classes
▪ I am not distracted at all	◯	◯
▪ It distracts me a little	◯	◯
▪ It distracts me some	◯	◯
▪ It distracts me a lot	◯	◯
▪ I cannot focus at all	◯	◯

**13. What type of distraction bothers you the most during class?**

▪	Online Classes	In Person Classes
▪ Visual distractions (i.e., Internet surfing)	◯	◯
▪ Audio distractions (i.e., alerts, music, conversations…)	◯	◯
▪ It does not bother me	◯	◯

**14. Have your professors introduced a course policy regarding the use of digital devices during class?**

▪	Online Classes	In Person Classes
▪ Yes	◯	◯
▪ No	◯	◯

**15. Which of the following statements best describes your use of digital devices for other than course-related activities?**

▪	Online Classes	In Person Classes
▪ I can use digital devices for reasons unrelated to school.	◯	◯
▪ It is my choice to use digital devices any time I want.	◯	◯
▪ I do not use digital devices during class because they distract me from learning.	◯	◯
▪ I cannot stop using digital devices during class even though I know they distract me.	◯	◯

**16. Do you think universities need to introduce policies that limit students’ use of digital devices during class?**

▪	Online Classes	In Person Classes
▪ Yes	◯	◯
▪ No	◯	◯

**17. Should instructors ban the use of digital devices during class?**

▪	Online Classes	In Person Classes
▪ Yes	◯	◯
▪ No	◯	◯

**18. What should an instructor do to a student who disturbs the class by using a digital device?**

▪	Online Classes	In Person Classes
▪ Talk to the student	◯	◯
▪ Ask the student to leave the class	◯	◯
▪ Confiscate the student’s digital device	◯	◯

**19. What measures should be taken against a student who uses digital devices during class for reasons unrelated to school?**

▪	Online Classes	In Person Classes
▪ Warning after the first offense, and punishment after subsequent offenses.	◯	◯
▪ Punishment after each offense	◯	◯
▪ No measure should be taken	◯	◯

## References

[1] Clapp W.C., Gazzeley A. (2012). Distinct mechanism for the Impact of Distraction and Interruption on Working Memory in Aging. Neurobiol. Aging.

[2] Gazzaley A., Rosen L.D. (2016). The Distracted Mind: Ancient Brains in a High-Tech World.

[3] Carrier L.M., Cheever N.A., Rosen L.D., Benitez S., Chang J. (2019). Multitasking across Generations: Multitasking Choices and Difficulty Ratings in Three Generations of Americans. Comput. Hum. Behav..

[4] Anderson J.Q., Rainie L. (2012). Millennials Will Benefit and Suffer due to Their Hyperconnected Lives, PEW Internet and American Project. https://www.pewresearch.org/internet/2012/02/29/millennials-will-benefit-and-suffer-due-to-their-hyperconnected-lives-2/.

[5] Brasel S.A., Gips J. (2011). Media Multitasking Behavior: Concurrent Television and Computer Usage. Cyberpsychol. Behav. Soc. Netw..

[6] Kessler S. (2011). 38% of College Students Can’t Go 10 Minutes without Tech. http://mashable.com/2011/05/31/college-tech-device-stats/.

[7] Nielsen (2010). Tech-or-Treat: Consumers Are Sweet on Mobile Apps. http://www.nielsen.com/us/en/insights/news/2014/tech-or-treat-consumers-are-sweet-on-mobile-apps.html.

[8] Ahonen T. (2012). Main Trends in the Telecommunications Market, MoMo Mobile Conference, Kiev. http://www.citia.co.uk/content/files/50_44-887.pdf.

[9] Harris Interactive (2011). Americans Work on Their Vacation, Including Checking Emails, Voicemails and Taking Calls. http://www.harrisinteractive.com.

[10] Hwang Y., Kim H., Jeong S.H. (2014). Why Do Media Users Multitask? Motives for General, Medium-Specific and Content-Specific Types of Multitasking. Comput. Hum. Behav..

[11] Wittmann B.C., Bunzeck N., Dolan R.J., Duzel E. (2007). Anticipation of Novelty Recruits Reward System and Hippocampus While Promoting Recollection. NeuroImage.

[12] Dwairy M., Dowell A.C., Stahl J.C. (2011). The Application of Foraging Theory to the Information Searching Behavior of General Practitioner. BMC Fam. Pract..

[13] Eastwood J.D., Frischen A., Fenske M.J., Smilek D. (2012). The Unengaged Mind Defining Boredom in Terms of Attention. Perspect. Psychol. Sci..

[14] Nieterman E., Zaza C. (2019). A Mixed Blessing? Students’ and Instructors’ Perspectives about Off-Task Technology Use in the Academic Classroom. Can. J. Scholarsh. Teach. Learn..

[15] Grinols A., Rajesh R. (2014). Multitasking with smartphones in the college classroom. Bus. Prof. Commun. Q..

[16] Hembrooke H., Gay G. (2003). The laptop and the lecture: The effects of multitasking in learning environments. J. Comput. High. Educ..

[17] Wurst C., Smarkola C., Gaffney M.A. (2008). Ubiquitous laptop usage in higher education: Effects on student achievement, student satisfaction, and constructivist measures in honors and traditional classrooms. Comput. Educ..

[18] Risko E.F., Buchanan D., Medimorec S., Kingstone A. (2013). Everyday attention: Mind wandering and computer use during lectures. Comput. Educ..

[19] Junco R., Cotton S.R. (2012). No A 4 U: The relationship between multitasking and academic performance. Comput. Educ..

[20] Beland L.-P., Murphy R. (2016). Ill communication: Technology, distraction & student performance. Labour Econ..

[21] Rosen L.D., Carrier L.M., Cheever N.A. (2013). Facebook and texting made me do it: Media-induced task-switching while studying. Comput. Hum. Behav..

[22] Lepp A., Barkley J.E., Karpinski A.C., Singh S. (2019). College Students’ Multitasking Behavior in Online versus Face-to-Face Courses. SAGE Open.

[23] Andrew-Gee E. (2015). Professors Push Back against Laptops in Lecture Hall. Globe & Mail. http://www.theglobeandmail.com/news/national/professors-push-back-against-laptops-in-the-lecture-hall/article26046828/.

[24] Teodorescu D., Aivaz K.A., Amalfi A. (2021). Factors affecting motivation in online courses during the COVID-19 pandemic: The experiences of students at a Romanian public university. Eur. J. High. Educ..

[25] Edu T., Negricea C., Zaharia R., Zaharia R.M. (2021). Factors influencing student transition to online education in the COVID 19 pandemic lockdown: Evidence from Romania. Econ. Res. Ekon. Istraž..

[26] Dumford A.D., Miller A.L. (2018). Online learning in higher education: Exploring advantages and disadvantages for engagement. J. Comput. High. Educ..

[27] Moreno M.A., Jelenchick L., Koff R., Eikoff J., Diermyer C., Christakis D.A. (2012). Internet use and multitasking among older adolescents: An experience sampling approach. Comput. Hum. Behav..

[28] Iluzada C.L., Wakefield R.L., Alford A.M. (2021). Personal Technology in the Classroom: Evaluating Student Learning, Attention, and Satisfaction. J. Eff. Teach. High. Educ..

[29] Flanigan A.E., Kiewra K.A. (2018). What College Instructors Can Do about Student Cyber-Slacking. Educ. Psychol. Rev..

[30] Morris P., Sarapin S. (2020). Mobile Phones in the Classroom: Policies and Potential Pedagogy. J. Media Lit. Educ..

[31] Migdalski S.T. (2017). Smartphone and Curriculum Opportunities for College Faculty. J. Learn. High. Educ..

[32] Aivaz K.-A., Teodorescu D. (2022). The Impact of the Coronavirus Pandemic on Medical Education: A Case Study at a Public University in Romania. Sustainability.

[33] Flanigan A.E., Babchuk W.A. (2022). Digital distraction in the classroom: Exploring instructor perceptions and reactions. Teach. High. Educ..

